# Diagnostic and therapeutic challenges of *Candida albicans* infection post-lumbar surgery in a rheumatoid arthritis patient: A case report and review of the literature

**DOI:** 10.1097/MD.0000000000042970

**Published:** 2025-07-25

**Authors:** Yulei Wang, Fanzhe Feng, Zhongzheng Yu, Jinlong Liang, Yongqing Xu, Tianhua Zhou, Nengqi Shao, Wenhao Xu, Yi Cui

**Affiliations:** a School of Clinical Medicine, Dali University, Dali, China; b Department of Orthopedics, 920th Hospital of Joint Logistics Support Force, Kunming, China.

**Keywords:** *Candida albicans*, discitis, lumbar infection, rheumatoid arthritis, spondylitis

## Abstract

**Rationale:**

Postoperative *Candida albicans* infections are exceedingly rare in urban populations, especially among patients on long-term immunosuppressive therapy. To date, there have been no reported cases of rheumatoid arthritis (RA) patients developing *C albicans* infections following spinal surgery. This report aims to highlight this rare occurrence and share insights on management strategies.

**Patient concerns:**

A 53-year-old Han Chinese male patient was admitted with severe pain in the right lower limb. He had a 3-year history of RA and was on continuous antirheumatic medication. The postoperative course was complicated by the development of a *C albicans* infection, which was confirmed after multiple diagnostic measures.

**Diagnoses:**

The patient was diagnosed with postoperative *C albicans* infection based on intraoperative sampling and laboratory confirmation 79 days after spinal surgery.

**Interventions:**

The patient underwent targeted antifungal therapy, including an 8-week intravenous course followed by a 3-month oral itraconazole regimen. Supportive measures included meticulous perioperative management, nutritional support, and physical rehabilitation.

**Outcomes:**

The infection was successfully controlled, leading to the complete resolution of symptoms. The patient achieved a clinical cure, demonstrating the efficacy of combined antifungal therapy and comprehensive perioperative care.

**Lessons:**

This case underscores the importance of vigilant perioperative management in RA patients, especially those with immunosuppression. Individualized treatment strategies, close monitoring of nutritional and functional status, and timely antifungal intervention are essential for preventing and managing such rare infections. Furthermore, a review of literature since 1980 has enhanced our understanding of risk factors, diagnosis, and treatment of postoperative *C albicans* infections, informing better clinical practice.

## 1. Introduction

Postoperative spinal infections are severe complications of spinal surgery, often warranting extended medication treatment or even numerous surgeries, significantly prolonging hospital stays and increasing medical and socioeconomic burdens.^[1,2]^ The pathogens responsible for these infections primarily include Gram-positive bacteria, Gram-negative bacteria, and fungi, with bacterial infections being prevalent. Early diagnosis and therapy typically simplify and standardize the treatment process for bacterial infections.^[3]^ In contrast, infections caused by fungi including *Candida albicans* present additional diagnostic challenges due to their rarity and complex pathogenesis.^[4]^ Improper treatment may cause spinal instability, neurological damage (including paralysis), or even death.^[3]^ Clinical treatment reports on RA patients with *C albicans* spinal infections post-surgery are currently unavailable.

Herein, we report a case of a patient with rheumatoid arthritis (RA) who developed a *C albicans* infection 79 days after lumbar spine surgery. The patient had been on disease-modifying antirheumatic drugs (DMARDs) for 5 years. Initial treatment with micafungin for 2 weeks did not yield significant improvement. Subsequently, the patient was administered a 6-week course of caspofungin followed by a 3-month oral itraconazole regimen successfully eradicating the infection. Moreover, we reviewed and compared the diagnosis and treatment of this newly presented case with postoperative *C albicans* infections in lumbar surgery cases from existing literature.

## 2. Case

**Figure 1.**
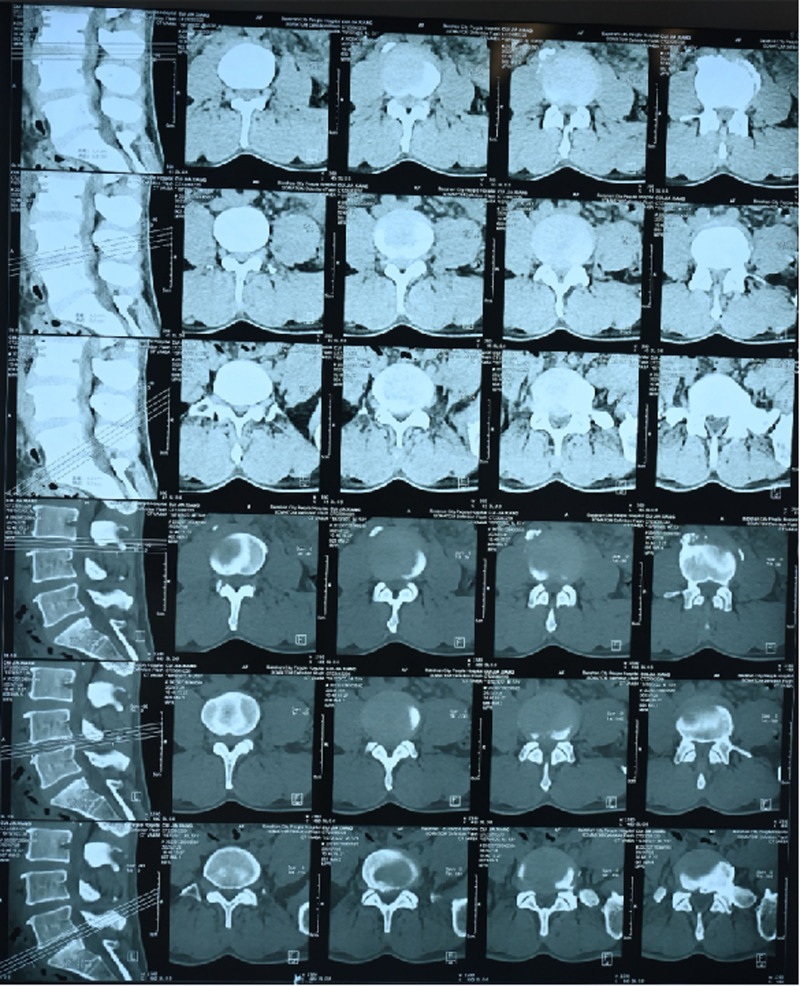
Preadmission computed tomography of the patient shows L4/5 and L5/S1 right-sided disc herniation.

We present the case of a 53-year-old Han Chinese male courier with a 30-year history of lower back pain and a 3-year history of right lower limb pain; the patient developed worsening symptoms over the past 2 weeks and was admitted for treatment. The patient has a 7-year history of RA and has been on methotrexate (MTX, 70 mg/wk) and leflunomide (10 mg/d) for 5 years. On physical examination, the patient had right leg claudication after walking 100 m and showed decreased sensation in the posterolateral right calf and the dorsum and sole of the right foot. We observed a positive straight leg raise test on the right side. A Lumbar CT scan revealed L4/5 and L5/S1 disc herniations leading to lumbar canal stenosis from L4 to S1 (Fig. 1). The patient underwent L4 to S1 open transforaminal lumbar interbody fusion surgery and was later discharged in improved condition.

**Figure 2.**
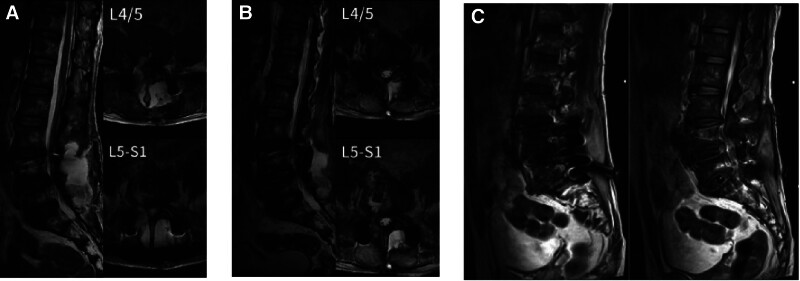
Compared to image (A), the high-signal intensity in the posterior paraspinal soft tissue in image (B) is significantly reduced. In image (C), high-signal intensity is observed in the intervertebral space and screw tracts.

The patient was readmitted with a fever 6 days after discharge. Physical examination revealed a temperature of 38.3°C and marked restriction in spinal mobility. MRI showed postoperative soft tissue edema from L4 to S1 (Fig. 2A). Laboratory test reports showed increased white blood cell count (WBC) at 9.34 × 10⁹/L, erythrocyte sedimentation rate (ESR) at 107 mm/h, and C-reactive protein (CRP) at 86.9 mg/L. A postoperative infection was suspected and we performed an urgent lumbar debridement. During the procedure, an inflow-outflow lavage system was placed bilaterally beside the pedicle screws, and soft tissue samples were collected for culture. The culture results indicated the growth of Burkholderia cepacia. Based on susceptibility testing, we treated the infection with targeted antibiotics and large volumes of normal saline (6000 mL/d/side) for wound irrigation. After 15 days of lavage, 3 consecutive cultures of the drainage fluid showed no bacterial growth, before removing the drains.

Nonetheless, 25 days after initial debridement, the patient remained febrile, with persistently increased infection markers. MRI continued to show soft tissue edema and fluid accumulation at L4 to S1 yet somewhat improved unlike that of previous imaging; significant fluid retention was still observed (Fig. 2B). These findings suggested ongoing infection, prompting a second debridement and continued therapy with sensitive antibiotics and saline lavage.

The patient still exhibited fever, and infection markers remained high (WBC 6.74 × 10⁹/L, ESR 119 mm/h, CRP 51.8 mg/L)48 days after the 2nd debridement. Follow-up MRI showed significantly high signal intensity at the screw tracts and intervertebral spaces (Fig. 2C). At this stage, we suspected that the infection originated from the implants, prompting us to perform a posterior lumbar debridement and removal of the spinal fixation screws, along with reinstallation of the inflow–outflow lavage system. Soft tissue cultures and metagenomic analysis confirmed *C albicans* growth. Susceptibility testing revealed sensitivity to amphotericin B, intermediate sensitivity to nystatin, miconazole, and itraconazole, and resistance to econazole, ketoconazole, and fluconazole. After a few days, *C albicans* was also detected in the sputum and wound drainage fluid of the patient, showing intermediate sensitivity to nystatin and resistance against all other tested agents.

**Figure 3.**
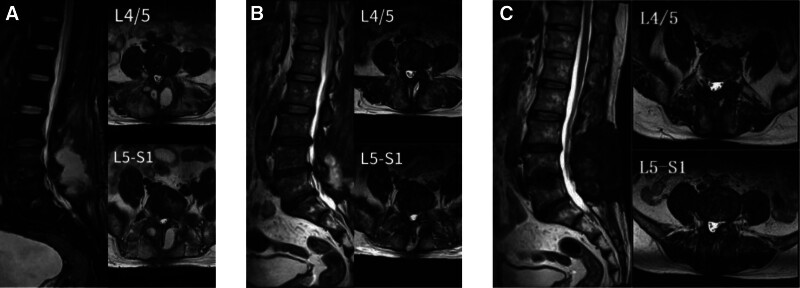
Images (A) and (B) show high-signal intensity in the edematous soft tissue of the posterior L4–S1 vertebral region; compared to image (A), the high-signal intensity in the posterior soft tissue in image (B) is significantly reduced, and high-signal intensity is also seen in the L4/5 and L5/S1 intervertebral spaces in image (B). In image (C), there is no obvious high-signal intensity in the posterior paraspinal soft tissue.

We started antifungal treatment with micafungin (150 mg daily) for 2 weeks; however, the patient continued to experience low-grade fever, and infection markers did not significantly improve (WBC 11.48 × 10⁹/L, ESR 108 mm/h, CRP 73 mg/L). A subsequent MRI showed persistent high signal intensity in the L4/5 and L5/S1 intervertebral spaces and surrounding soft tissues (Fig. 3A). At this juncture, we faced a critical decision regarding the removal of the interbody fusion cage. During this period, we also observed the poor nutritional status of the patient and recommended increased protein and calorie intake, as well as encouraging daily ambulation and functional exercise. After the recommendations from infectious disease specialists, we transitioned to a 6-week regimen of caspofungin (50 mg intravenous injection daily) and levofloxacin (500 mg orally daily).

After 6 weeks of treatment, the infection markers significantly decreased (WBC 6.28 × 10⁹/L, ESR 45 mm/h, CRP 17.3 mg/L), and body temperature stabilized below 37.5°C; besides, follow-up MRI showed substantial absorption of the soft tissue fluid (Fig. 3B). Considering the resistance to multiple azole antifungals, we administered a subsequent 3-month course of oral itraconazole (200 mg daily). At the 3-month follow-up, the MRI showed no substantial soft tissue edema (Fig. 3C); infection markers returned to normal levels.

## 3. Literature review

A comprehensive review of case reports and literature on *C albicans* infections after spinal surgery was carried out using the search terms “*C albicans*,” “vertebral osteomyelitis,” “spinal osteomyelitis,” and “spondylodiscitis.” This search was performed in databases including PubMed and Google Scholar, covering publications since 1980. A total of 10 cases of *C albicans* infection postspinal surgery. Our review focused on the risk factors of patients, surgical methods, infection sites, diagnostic intervals, pharmacological treatment strategies, treatment durations, and outcomes (refer to Table 1 for details).^[5–10]^

**Table 1:** Cases of *Candida albicans* infection postspinal surgery.

Author	Year	Age	Gender	Risk factors	Surgical method	Segment	Time to diagnosis after initial surgery	Clinical manifestation	Drug treatment	Treatment duration	Final outcome
Adhikari P, et al^[5]^	2023	61	Male	Pyriform sinus carcinoma, tuberculosis	Anterior C5–C6 corpectomy and cage placement	C5–C7	28 d	Not mentioned	Voriconazole	12 mo	Cured
CS CSSZ FC and Lim H^[6]^	2023	67	Female	Diabetes, sepsis, lumbar artery pseudoaneurysm	T12–L5 PSO correction	T12–L5	Not mentioned	Not mentioned	Not mentioned	Not mentioned	Cured
Upadhyay A, et al^[7]^	2020	61	Male	Not mentioned	L4/5 discectomy	L4–5	42 d	Back pain	Amphotericin B, itraconazole	3 mo	Cured
Zou MX, et al^[8]^	2015	71	Male	Not mentioned	L4–L5 discectomy	L3–L5	23 d	Severe back pain	Amphotericin B	7 mo	Cured
		56	Female	Not mentioned	L3–L4 discectomy	L3–L4	100 d	Severe back pain	Amphotericin B	6.5 mo	
		59	Male	Not mentioned	L4–L5 microdiscectomy	L3–S1	25 d	Severe back pain	Amphotericin B	6 mo	
Brembilla C, et al^[9]^	2014	27	Male	Neck trauma, long-term antibiotic use	Anterior C5–C6 plating and cage placement	C4–C7	120 d	Neck pain	Fluconazole	12 mo	Cured
Parry MF, et al^[10]^	2001	19	Female	Surgical personnel with artificial nails	L4–5 discectomy	L4–L5	54 d	Back pain, muscle spasms	Amphotericin B, fluconazole	13.5 mo	Cured
		37	Male	Surgical personnel with artificial nails	L4–5 discectomy	L4–L5	44 d	Back pain and persistent numbness in the right lower limb	Amphotericin B, 5-fluorocytosine, fluconazole	12 mo	
		64	Male	Surgical personnel with artificial nails	L4–5 discectomy	L4–L5	83 d	Back pain, left leg, and hip pain	Amphotericin B, fluconazole	13 mo	

PSO = pedicle subtraction osteotomy.

A total of 59 relevant cases were identified in our extensive search. We eventually focused on only 10 cases of postoperative *C albicans* infections of the spine, paying special attention to patient risk factors, surgical methods, infection sites, diagnostic intervals, treatment strategies, treatment durations, and outcomes. The results showed that the patients had an average age of 52.2 years (ranging from 19 to 71 years), with 7 males (70%) and 3 females (30%). Risk factors included cancer, tuberculosis, diabetes, surgical site trauma, long-term antibiotic use, and wearing jewelry by surgical personnel. The infection sites involved the cervical spine in 2 cases (20%) and the lumbar spine in 8 cases (80%). The time from initial surgery to diagnosis ranged from 23 to 120 days. Postoperative symptoms were majorly low back pain and lower limb pain or numbness. The treatment drugs included voriconazole, amphotericin B, and itraconazole, with treatment durations ranging from 3 months to 13.5 months, averaging 9.44 months. All patients were ultimately cured.

## 4. Discussion

This case report describes a patient with a 7-year history of RA who has been under long-term oral DMARDs. We also sought to establish whether this increases the risk of *C albicans* infection after spinal surgery, a topic that warrants further discussion. RA is a common systemic inflammatory joint disease, with existing studies reporting that RA patients face a higher risk of infection during the perioperative period.^[11–13]^ Current literature often reports *C albicans* infections after joint surgeries. For instance, Springer described a case involving a patient with a 30-year RA history who developed a *C albicans* infection after undergoing a shoulder replacement while on long-term MTX and leflunomide treatment. The authors attributed the infection to prolonged immunosuppression from several drugs.^[14]^ Similarly, Luo reported a case of a patient with RA who developed a *C albicans* infection after total knee replacement.^[15]^ Here, the patient stopped MTX and leflunomide medication 45 days after lumbar surgery, and *C albicans* was cultured 79 days post-surgery, with an interval of only 34 days between the 2 events. Notably, the time frame between initial surgery and the diagnosis of *C albicans* infection ranges from 23 to 120 days.^[5–10]^ Furthermore, the risk factors for *C albicans* infection include long-term use of broad-spectrum antibiotics, central venous access, immunosuppression, neutropenia, chronic granulomatous disease, and intravenous drug use.^[16]^ As such, we hypothesize that the 7-year history of RA and 5-year use of DMARDs, which resulted in an immunosuppressed state are substantial risk factors for developing *C albicans* infection. Moreover, the prolonged use of multiple antibiotics post-surgery might have also contributed to the infection.

Diagnosis of *C albicans*-induced spinal infection relies on clinical presentation, imaging, and laboratory tests. However, a definitive diagnosis requires biopsy through puncture or surgical tissue sampling.^[17]^ Typical symptoms of spinal infection include back pain, neurological deficits, and increased infection markers. Fungal infections postspinal surgery often lack specific clinical signs, with surgical site pain being the most common presentation. In cases reported by Carlo and colleagues, severe neck pain was noted^[9]^; Parry et al reported varying degrees of back and lower limb pain in 3 cases.^[10]^ Of note, MRI is the preferred imaging modality for diagnosing spinal discitis, as it can reveal early erosive bone changes.^[17,18]^ Laboratory tests often show increased ESR and CRP in most fungal infections; on the other hand, white blood cell counts may not be significantly elevated.^[12,19]^ Increased procalcitonin levels typically indicate bacterial infections, but do not exclude fungal infections.^[20]^ In this case, the patient initially presented with Burkholderia cepacia infection postoperatively, which masked the presence of *C albicans*. The rarity of *C albicans* infections contributed to our initial oversight, leading to its confirmation only on the 79th day through surgical tissue cultures.

Treatment of Candida-induced spinal infections includes both pharmacological and surgical approaches. According to the 2016 guidelines from the Infectious Diseases Society of America, confirmed Candida infections should be treated with fluconazole (6 mg/kg daily for 6–12 months) or initially with an echinocandin (e.g., micafungin, 100 mg daily) for at least 2 weeks, followed by fluconazole (6 mg/kg daily for 6 –12 months).^[21]^ Huang reported a case of *C albicans* infection at the C4 to T1 segments, successfully treated with 14 days of intravenous micafungin followed by 6 to 12 months of 400 mg daily oral fluconazole.^[22]^ Nevertheless, treatment regimens can vary; Joshi documented a case treated with 7 days of caspofungin, followed by 6 months of fluconazole, leading to a successful resolution.^[23]^ In the present case, we switched to 6 weeks of caspofungin (50 mg daily) after 2 weeks of micafungin treatment with no significant improvement, resulting in significant patient improvement. This was followed by 3 months of maintenance treatment with oral itraconazole, resulting in a successful cure. Therefore, we hypothesize that treatment plans should be customized to suit an individual condition, particularly for those with poor infection control, who might require prolonged echinocandin treatment. Treatment should transition to oral medications once the condition of the patient stabilizes, body temperature remains below 37.5°C, and infection markers return to near-normal levels; this ensures efficacy while potentially reducing the overall treatment duration. Nonetheless, this hypothesis warrants further validation due to the limited number of cases.

In some cases, surgical debridement is also essential. The primary objectives include removing infected tissues, reducing inflammatory progression, providing sufficient blood supply to promote healing, and maintaining or restoring spinal stability.^[24]^ Surgical indications include neurological deficits, spinal cord compression, persistent spinal instability, large epidural abscesses, or severe intractable pain that fails to respond to appropriate antimicrobial therapy.^[12]^ Brembilla et al reported a patient with persistent neck pain for 6 months post-cervical surgery; a CT scan showed extensive bone destruction and almost complete anterior displacement of the implants, causing loss of cervical stability, hence requiring complete removal of the implants.^[9]^ In our case, MRI showed high signal intensity in the pedicle screw tracts and intervertebral spaces; we observed no signs of bone destruction or spinal instability. Therefore, we opted for posterior debridement and removal of the pedicle screws, while preserving the interbody fusion cage. Subsequent antifungal treatment resulted in a successful outcome.

## 5. Conclusion

This case report presented an unprecedented *C albicans* infection in an RA patient after spinal surgery. Fungal infections after spinal surgery often present themselves without specific symptoms. Therefore, a high suspicion of fungal infection should be maintained in cases where conventional antibiotic therapy is ineffective. Besides, further confirmation should be sought through biopsy and culture. After diagnosing *C albicans* infection, immediate antifungal treatment should be initiated, along with supportive measures to improve nutrition and promote functional exercise. If surgical intervention is necessary, comprehensive assessment and planning of the surgical approach are critical. This case underscores the importance of appropriately managing antirheumatic medications during the perioperative period and highlights the treatment strategies for *C albicans* infection after spinal surgery.

## Acknowledgments

We thank the following individuals for their guidance in actively treating the patient: Fanzhe Feng, Zhongzheng Yu, Jinlong Liang, Yongqing Xu, and Tianhua Zhou. We also thank Nengqi Shao and Wenhao Xu for collecting previous case studies. Special thanks to Yi Cui for the support provided throughout the manuscript writing process.

## Author contributions

**Conceptualization:** Nengqi Shao, Wenhao Xu.

**Data curation:** Yulei Wang, Zhongzheng Yu.

**Formal analysis:** Fanzhe Feng.

**Funding acquisition:** Yongqing Xu.

**Investigation:** Yulei Wang, Zhongzheng Yu.

**Methodology:** Yi Cui.

**Validation:** Jinlong Liang, Yongqing Xu, Tianhua Zhou.

**Visualization:** Jinlong Liang.

**Writing – original draft:** Yulei Wang.

**Writing – review & editing:** Yi Cui.
